# Plasma Organochlorines and Subsequent Risk of Prostate Cancer in Japanese Men: A Nested Case–Control Study

**DOI:** 10.1289/ehp.0901214

**Published:** 2009-12-17

**Authors:** Norie Sawada, Motoki Iwasaki, Manami Inoue, Hiroaki Itoh, Shizuka Sasazuki, Taiki Yamaji, Taichi Shimazu, Shoichiro Tsugane

**Affiliations:** Epidemiology and Prevention Division, Research Center for Cancer Prevention and Screening, National Cancer Center, Tokyo, Japan

**Keywords:** Japanese men, JPHC Study, nested case-control study, organochlorines, prostate cancer

## Abstract

**Background:**

Although accumulating evidence suggests that exposure to organochlorine pesticides and polychlorinated biphenyls (PCBs) may contribute to the development of prostate cancer, few investigations have used biological samples to classify exposure to specific organochlorines. To our knowledge, this is the first prospective study to investigate the association between blood levels of organochlorines and prostate cancer risk.

**Methods:**

We conducted a nested case–control study using data from the Japan Public Health Center–based Prospective (JPHC) Study. A total of 14,203 men 40–69 years old who returned the baseline questionnaire and who provided blood samples were followed from 1990 to 2005. Using a mean follow-up period of 12.8 years, we identified 201 participants who were newly diagnosed with prostate cancer. Two matched controls for each case were selected from the cohort. We used a conditional logistic regression model to estimate the odds ratios (ORs) and 95% confidence intervals (CIs) for prostate cancer in relation to plasma levels of nine organochlorines: PCBs, dichlorodiphenyltrichloroethane (DDT), hexachlorobenzene (HCB), β-hexachlorocyclohexane (β-HCH), *trans*- and *cis*-nonachlor, oxychlordane, and mirex.

**Results:**

No statistically significant association with total prostate cancer was seen for any plasma organochlorine, although we did observe an insignificant inverse association for plasma HCB and β-HCH. Total PCB in plasma was also inversely associated with advanced prostate cancer but without statistical significance.

**Conclusion:**

Our results suggest that no overall association exists between prostate cancer and organochlorines at the levels measured in our study population.

The age-adjusted incidence of prostate cancer in Japan has increased over the past two decades (Center for Cancer Control and Information Services 2007). One cause is considered to be screening for prostate-specific antigen (PSA), but other causes cannot be ruled out. The etiology of prostate cancer is poorly understood, but established risk factors include increasing age, African-American ethnicity, and a positive family history of prostate cancer (Adami 2008). Hormonal factors may play a role in the etiology of prostate cancer, because androgens, particularly testosterone and dihydrotestosterone, are essential to the normal growth and functioning of the prostate (Adami 2008). For this reason, attention has been focused on the possible role of organochlorines, such as dichlorodiphenyltrichloroethane (DDT) and polychlorinated biphenyls (PCBs), which are endocrine disruptors that may have adverse effects on the male reproductive system (Safe 2000).

Most evidence to date suggests that farming (Blair et al. 1992; Buxton et al. 1999; Cerhan et al. 1998; Dich and Wiklund 1998; Fleming et al. 1999; Inskip et al. 1996; Keller and Howe 1994; Krstev et al. 1998; Meyer et al. 2007; Morrison et al. 1993; Parker et al. 1999; Settimi et al. 2001; Sharma-Wagner et al. 2000; Van Der Gulden and Vogelzang 1996; van der Gulden et al. 1995) and pesticide application (Dich et al. 1997; Fleming et al. 1999) are associated with an increased risk or excess incidence and mortality of prostate cancer, although findings of reduced or nonexcess risk have also been reported (Boers et al. 2005; Fleming et al. 2003; Fritschi et al. 2007; Zeegers et al. 2004). Several meta-analyses of prostate cancer and occupational pesticide exposure, including those in farming (Acquavella 1999; Keller-Byrne et al. 1997), any occupation-related pesticide exposure (Van Maele-Fabry and Willems 2003), pesticide application (Van Maele-Fabry and Willems 2004), and pesticide manufacture (Van Maele-Fabry et al. 2006), have reported an increased risk in workers engaged in these occupations. Taken together, these studies suggest that organochlorine exposure may play an important role in the development of prostate cancer. However, one study compared the expert-assessed likelihood of pesticide exposure based on detailed, individual-specific questionnaire and job-specific module interview information with reported farm-related job titles as a surrogate for pesticide exposure. The authors reported that farming job title alone is a poor indicator of the likelihood of pesticide exposure, as classification of all farm jobs as pesticide exposed is likely to substantially overestimate the number of individuals exposed (Macfarlane et al. 2009). On this basis, exposure assessment in some studies may have been insufficient. Moreover, few studies have used biological samples (Hardell et al. 2006; Ritchie et al. 2003, 2005) or investigated the association between prostate cancer and specific organochlorines (Alavanja et al. 2003; Charles et al. 2003; Prince et al. 2006; Settimi et al. 2003). Evaluation of blood levels of individual organochlorines should therefore provide new information on their association with prostate cancer.

In Japan, although the use of organochlorine compounds was banned in the first half of the 1970s, their long half-lives have resulted in the identification of residues in several foods and an ongoing presence in the blood or breast milk (Hanaoka et al. 2002; Konishi et al. 2001b; Lordo et al. 1996). Nevertheless, the association between organochlorines and prostate cancer in the general population is not clearly understood.

In this study, we investigated the association between plasma organochlorine level and prostate cancer in a nested case–control study within a large prospective cohort study.

## Materials and Methods

### Study population

The Japan Public Health Center–based Prospective (JPHC) Study was initiated in 1990 for cohort I and in 1993 for cohort II. Cohort I consisted of five public health center (PHC) areas (Iwate, Akita, Nagano, Okinawa, and Tokyo) and cohort II included six PHC areas (Ibaraki, Niigata, Kochi, Nagasaki, Okinawa, and Osaka) in Japan. The study design has been described in detail elsewhere (Tsugane and Sobue 2001). The study population was defined as all residents 40–59 years old in cohort I and 40–69 years old in cohort II at the start of the respective baseline survey.

In the present analysis, we excluded the Tokyo subjects from the analyses because data on cancer incidence were not available. Thus, after the exclusion of ineligible subjects (*n* = 144), we had identified a population-based cohort of 65,657 men. Informed consent was obtained from each participant implicitly when they completed the baseline questionnaire in which the purpose of the study and the follow-up methods were described. This study was approved by the Institutional Review Board of the National Cancer Center, Tokyo, Japan.

### Questionnaire survey

A baseline self-administered questionnaire survey was conducted in 1990–1994. The questionnaire was distributed mostly by hand and partly by mail, and incomplete answers were supplemented by telephone interview. A total of 50,435 men (response rate 77%) returned the questionnaire, which included smoking and drinking habits, current height (whole centimeters), body weight (kilograms), occupation, personal and family medical history, and diet. The questionnaire inquired about a medical history of cancer, cardiovascular disease, diabetes mellitus, and other diseases (Konishi et al. 2001a). For dietary assessment, we used data from the validated food frequency questionnaires found in the baseline survey for cohorts I and II, which included 44 and 52 food items, respectively (Tsubono et al. 1996).

### Blood collection

Subjects voluntarily provided 10 mL of blood during health checkups in 1990–1995. Blood samples were divided into plasma and buffy layers and preserved at −80°C until analysis. Among respondents to the baseline questionnaire, a total of 14,203 men (28%) donated blood.

### Follow-up

Subjects were followed from the baseline survey until 31 December 2005. Changes in residence status, including the residence status of participants who were still living, were identified annually through the residential registry in their PHC area. Of the study subjects, 749 (5.3%) moved out of the study area, and 28 (0.2%) were lost to follow-up.

### Selection of cases and controls

Study staff identified incidence data on prostate cancer by using spontaneous reporting from major local hospitals in the study area to ascertain cases and by linking data from population-based cancer registries, with permission from the local governments responsible for the registries. Death certificates were used to supplement the information on cancer incidence. Cases were coded using the *International Classification of Diseases for Oncology* (World Health Organization 2000). Up to the end of the study period after blood collection, we identified 201 newly diagnosed prostate cancer cases among 14,203 men who returned the baseline questionnaire, who reported no history of prostate cancer, and who provided blood samples. Of these, 97% of cases were pathologically confirmed, and 0.5% were confirmed by death certificate only. We defined advanced cases by a diagnosis of extraprostatic or metastatic cancer involving lymph nodes or other organs at first diagnosis of prostate cancer. If this information was not available, advanced cases were defined as those with a high Gleason score (8–10) or poor differentiation. These criteria were selected to identify advanced cases with a high likelihood of poor prognosis. For the remaining cases, the cancer had not metastasized to other organs. We identified a total of 48 men with advanced cancer and 144 with localized cancer; for 9 (4% of total) cases, the cancer stage was undetermined.

For each case, two controls were selected from among subjects with no history of prostate cancer when the case was diagnosed. Controls were matched for each case by age (within 3 years), PHC area, residence (city or town and village), date on which blood was obtained (within 60 days), time of day of blood collection (within 3 hr), and duration of fasting at blood collection (within 3 hr).

### Laboratory assays

We measured the following plasma organochlorines: *o,p*′-dichlorodiphenyltrichloroethane (*o,p*′-DDT), *p,p*′-DDT, *p,p*′-dichlorodiphenyldichloroethylene (*p,p*′-DDE), hexachlorobenzene (HCB), β-hexachlorocyclohexane (β-HCH), *trans*-nonachlor, *cis*-nonachlor, oxychlordane, and mirex, and > 41 PCB congeners (International Union of Pure and Applied Chemistry numbers 17, 28, 51, 52/69, 43/49, 48/47, 44, 74, 66, 77, 90/101, 99, 123, 118, 114, 105, 126, 146, 153, 164/163, 138, 128/162, 167, 156, 169, 182/187, 183, 174, 177, 180, 170, 189, 202, 201, 198/199, 196, 203, 194, 208, 206, and 209). Extraction and assay of these compounds from 500 μL plasma samples were conducted at Shimadzu Techno Research Inc. (Kyoto, Japan) using the modified method previously described (Itoh et al. 2009). Organochlorines and PCBs were detected and measured using a high-resolution mass spectrometer (Autospec Ultima, Waters/Micromass, Manchester, UK) with selected ion monitoring connected to a gas chromatograph (HP6890, Hewlett Packard, Palo Alto, CA, USA) equipped with a capillary column (HT8-PCB fused silica capillary column 60 m × 0.25 mm i.d. for PCBs; DB-17HT fused silica capillary column 30 m × 0.32 mm i.d., 0.15 μm for the organochlorines) based on isotope-dilution mass spectrometry. Limits of detection (LODs) were 3.0 pg/g wet for organochlorine pesticides and 2 pg/g wet for PCB congeners. Measurement values < LODs were recorded as zero in this study. When we recorded these undetectable values as the LODs or as half the LOD values, the results were not significantly changed. The sum of PCBs (total PCBs) was based on the detectable values only. Based on average values for each PCB congener, total PCBs among control participants consisted of the following PCB congeners: 153 (22.3%), 180 (15.9%), 138 (11.2%), 182/187 (7.0%), 118 (5.3%), 170 (4.5%), 164/163 (4.5%), 99 (3.2%), 74 (2.9%), 146 (2.8%), 194 (2.3%), 198/199 (2.3%), 156 (1.9%), 183 (1.7%), 48/47 (1.4%), 177 (1.3%), 203 (1.1%), 105 (1.1%), and the remaining PCBs. The remaining PCBs are not shown because their values were < 1.0%. Laboratory precision in this measurement was assessed using blank and quality control samples in the same batch at each assay. Interbatch coefficients of variation ranged between 2% and 8% based on 24 replicated measurements of plasma samples at a mean concentration (picograms per gram wet) range of 3.6 (*cis*-nonachlor) to 688 (*p,p*′-DDE). All analyses showed high reliability, with an intraclass correlation of > 0.96. Cases and matched controls were assayed in the same batch. All samples were analyzed by laboratory analysts blinded to case–control status.

Total cholesterol and triglycerides were enzymatically measured at Kyoto Biken Laboratories Inc. (Kyoto, Japan) to examine organochlorine levels relative to the amount of lipid in plasma. Lipid-adjusted concentrations of organochlorines were calculated by dividing plasma organochlorine concentrations by total plasma lipid concentrations. We used the method described by Phillips et al. (1989) to estimate total plasma lipids.

### Statistical analysis

We evaluated baseline characteristics and plasma organochlorine levels between cases and controls by the Mantel-Haenszel test using matched pair strata. Odds ratios (ORs) and 95% confidence intervals (CIs) for prostate cancer risk were estimated by quartile level of plasma organochlorine using a conditional logistic regression model. Because distributions of all organochlorine concentrations in plasma are positively skewed, the values were transformed to the log scale. Quartile cutoff points were based on the frequency distribution of controls. Organochlorine levels were analyzed using both the crude and lipid-adjusted plasma values, but because conclusions were similar, we show only the results of the lipid-adjusted analyses. ORs and 95% CIs were adjusted for the following variables as potential confounders collected at baseline: smoking status (never, former, and current), alcohol intake (almost never, 1–3 times/month, ≥ 1 time/week), body mass index (< 22, 22–23.9, ≥ 24 kg/m^2^), marital status (yes/no), intake of miso soup (< 3 bowls/week, 1 bowl/day, 2 bowls/day, ≥ 3 bowls/day), and green tea intake (< 1 cup/day, 1–2 cups/day, 3–4 cups/day, ≥ 5 cups/day). These variables are either known or suspected risk factors for cancer or had previously been associated with the risk of prostate cancer (Kurahashi et al. 2007, 2008a). A family history of prostate cancer was not evaluated as a potential confounding factor because only one case subject reported it. Linear trends for OR were tested in the conditional logistic regression model using the median values in each category. All *p*-values are two-sided, and statistical significance was determined at *p* < 0.05. Additionally, we estimated the ORs of prostate cases stratified by stage as well as those for all cases. All statistical analyses were performed with SAS software (version 9.1, SAS Institute, Inc., Cary, NC, USA).

## Results

Basic characteristics of case subjects and matched controls at baseline are shown in Table 1. Cases tended to smoke less and to consume more green vegetables. The proportion of daily consumers of green tea, miso soup, milk, fish, and pork did not differ between cases and controls. Although PSA screening information was available for only 78% of cases, the proportion of screening-detected cancers was 52.6% (82 cases), of which 87.8% (72 cases) were diagnosed as localized cases (data not shown).

Table 2 shows that no significant differences were found between cases and controls in median plasma levels of organochlorines. *p,p*′-DDT, *p,p*′-DDE, HCB, β-HCH, *trans*-nonachlor, *cis*-nonachlor, oxychlordane, and mirex were detected in plasma in all subjects, but some subjects were < LOD in *o,p*′-DDT and 17 PCB peaks: the percentages of cases and controls with levels below the detection limit were 0% and 0.5% for *o,p*′-DDT, respectively, and from 0.5% for PCB 66 to 99% for PCB 77, and 0.2% for PCB 90/100 to 100% for PCB 77, respectively.

Table 3 shows ORs and 95% CIs of total prostate cancer risk according to organochlorine levels in plasma. No association between plasma levels of any organochlorine and total prostate cancer was observed, although ORs in HCB and β-HCH were below unity, with ORs for the highest versus lowest quartile of 0.52 (95% CI, 0.21–1.25; *p-*trend = 0.11) and 0.56 (95% CI, 0.30–1.01; *p-*trend = 0.05), respectively. A significantly decreased relative risk of prostate cancer was observed for the second quartile group of mirex (OR = 0.54; 95% CI, 0.30–0.97), but the OR for the highest group was above 1. The results were not significantly changed when further adjustment was made for other potential confounders such as dietary intake of milk, vegetables, fish, and meat. Further, the results did not substantially change when we excluded subjects who provided a nonfasting blood sample, that is, within 6 hr after a meal. Additionally, no material changes were seen when subjects were restricted to those without occupational exposure to organochlorines or to those detected by screening, or when they were stratified by year of diagnosis (up to 1997 and 1998 or later).

We next classified the data according to prostate cancer stage (Table 4). No significant associations were seen between any organochlorine and localized prostate cancer, although adjusted ORs in second and third quartile groups for *o,p*′-DDT were insignificantly elevated at 1.79 (95% CI, 0.88–3.65) and 2.01 (95% CI, 0.96–4.22), respectively. In contrast, the OR in the third group for total PCBs was significantly decreased for advanced prostate cancer risk at 0.18 (95% CI, 0.04–0.92), although the trend was not statistically significant (*p-*trend = 0.17). The other organochlorines showed no statistically significant association with advanced prostate cancer.

We also analyzed associations for 41 individual PCB congeners but found none. Moreover, no association was found when the PCB congeners were grouped according to the method of Wolff et al. (1997) based on structural and biological activity: no association was seen for any group, with ORs (95% CIs) for the highest versus lowest quartile of 1.37 (0.75–2.50) for group 1A, 1.27 (0.63–2.58) for group 1B, 1.11 (0.57–2.18) for group 2A, 1.13 (0.60–2.14) for group 2B, and 1.06 (0.54–2.09) for group 3 (data not shown).

## Discussion

Our results showed that the plasma level of organochlorines was not positively associated with incident prostate cancer among Japanese men. This study, which to our knowledge is the first prospective investigation of plasma levels of organochlorines and prostate cancer, suggests that exposure to organochlorines is not associated with prostate cancer in general populations.

Many epidemiologic studies have indicated that organochlorine exposure may increase the risk or cause an excess incidence and mortality of prostate cancer. Several meta-analyses have reported statistically significant associations, with relative risk estimates for prostate cancer in farmers versus general populations of 1.08 (95% CI, 1.06–1.11) (Blair et al. 1992) and 1.12 (95% CI, 1.01–1.24) (Keller-Byrne et al. 1997) and in the presence versus absence of exposure to occupational pesticide of 1.13 (95% CI, 1.04–1.22) (Van Maele-Fabry and Willems 2003), 1.24 (95% CI, 1.06–1.45) (Van Maele-Fabry and Willems 2004), and 1.28 (95% CI, 1.05–1.58) (Van Maele-Fabry et al. 2006). Several experimental findings have shown that organochlorine pesticides are endocrine disruptors that may have adverse effects on the male reproductive system (Safe 2000). These various findings have led to the hypothesis that exposure to some organochlorine compounds may increase risk of prostate cancer. Our results do not support this hypothesis, however. This discrepancy in results between the general population in our study and farmers and pesticide users in several meta-analyses may be explained by the following factors. First, it is possible that exposure in our study population was low relative to exposures in other study populations. For example, the median value of plasma lipid-adjusted DDE among black male farmers in North Carolina was higher (1,213 ng/g) (Martin et al. 2002) than among those in our populations (910 ng/g). Even if positive associations between organochlorines and prostate cancer do exist, the adverse effects may not be apparent at low levels. Second, workers who work with organochlorines might also be exposed to other potential chemicals, such as dioxin, which was not measured in our study. In a meta-analysis (Van Maele-Fabry et al. 2006), workers engaged in manufacturing of phenoxy herbicides contaminated with dioxins and furans were shown to have an increased risk of prostate cancer. (Chamie et al. 2008). Third, factors other than organochlorines may increase the risk of prostate cancer in farmers and workers who work with pesticides; for example, lifestyle factors such as dietary habits may be unique to particular occupations.

With regard to exposure to specific chemicals, a significantly increased relative risk for DDT was shown in a case–control study (Settimi et al. 2003) and in a prospective study (Alavanja et al. 2003). Although DDT mimics the actions of estrogen by binding to estrogen receptors, and *p,p*′-DDE has been demonstrated to be antiandrogenic (Kelce et al. 1995), plasma DDT and DDE levels in our study were not associated with prostate cancer. Meanwhile, exposure to PCB showed a positive association with prostate cancer mortality in a cohort of workers (Charles et al. 2003; Prince et al. 2006), and PCB levels in blood increased risk of prostate cancer in three studies (Hardell et al. 2006; Ritchie et al. 2003, 2005). In one study using serum samples, the multivariate ORs of prostate cancer among men with middle concentrations of oxychlordane and PCB 180 were more than three times those among men with the lowest concentrations, without a linear dose response (Ritchie et al. 2003). In a further analysis in the same data set, a substantially greater than doubling of the relative risk of prostate cancer was observed among men with the highest lipid-unadjusted concentration of PCBs in group 3 (Wolff’s method) compared with those with the lowest concentrations (Ritchie et al. 2005). The third study, which used adipose tissue, reported that PCB 153 in greater than median concentrations was positively associated, with OR of 3.15 (95% CI, 1.04–9.54) (Hardell et al. 2006). PCB congeners were classified as estrogenic, antiestrogenic, or enzyme-inducing groups based on structural, biological, and pharmacokinetic considerations (Wolff et al. 1997). Although PCB 153 and 180 are classified in group 3 and considered to be CYP1A1 and CYP2B inducers (Wolff et al. 1997), our present results showed no positive association between prostate cancer and any PCB congener. The reason for the difference between our present findings and these case–control study results using biological samples is not clear. Given our finding that the association with plasma total PCBs tended to differ by stage of prostate cancer, one possible reason might be differences in the proportion of localized and advanced prostate cancer cases among different studies. However, this would not explain the divergence, because the proportions of localized versus advanced prostate cancers in our study population was relatively high and consistent with those in other highly screened populations in which positive associations between PCB and prostate cancer have been reported (Ritchie et al. 2005). The divergence between these previous studies and our present study might be due to differences in the time samples were collected (around 2000 in the previous studies vs. around 1990 in our study) or age range of subjects (47–85 years old in the previous studies vs. 40–69 years old in our study), which might have led to differences in the variation of exposure.

HCB and β-HCH tended to be inversely associated with prostate cancer in our study, although without statistical significance. We also found a lack of association with plasma oxychlordane, *cis*- and *trans*-nonachlor, and mirex levels. In one of the case–control studies, high levels of HCB and *trans*-chlordane in adipose tissue were positively associated with prostate cancer risk (Hardell et al. 2006), whereas in an *in vitro* study, pesticides including β-HCH induced the progression of prostate cancer cells by activating protein tyrosine kinase (Tessier and Matsumura 2001). In contrast, oxychlordane, *cis*- and *trans*-nonachlor, β-HCH, and mirex exhibited no interaction with the androgen receptor in a reporter gene assay (Schrader and Cooke 2000). Results for associations of organochlorines other than DDT and PCB with prostate cancer are therefore inconsistent, and further studies are needed.

Given that the majority of studies investigating the association between organochlorines in blood and breast cancer have shown no association, the overall lack of association between plasma organochlorines and prostate cancer in our study is not surprising (Itoh et al. 2009; Iwasaki et al. 2008; Lopez-Cervantes et al. 2004). Low-level environmental organochlorine exposures experienced by general populations may not be associated with an increased risk of cancer. Further studies using biological samples are required not only in general populations, but also for those in specific occupations such as farmers, pesticide applicators, and pesticide-manufacturing workers; several previous studies have indicated that persons in these professions may have an increased risk of prostate cancer (Acquavella 1999; Keller-Byrne et al. 1997; Van Maele-Fabry and Willems 2003, 2004; Van Maele-Fabry et al. 2006).

Several limitations in the interpretation of our findings warrant consideration. First, the number of prostate cancer cases was relatively small, which reflects the low incidence rate in Japan (age-standardized rate per 100,000 world population: 20.7 in 2002) (Matsuda et al. 2008). Thus, the power to detect weak associations may have been insufficient. Nevertheless, our study size is relatively large compared with the previous studies that used biological samples. Second, our baseline exposure estimates, which occurred at an average age of 58 years, may not have captured etiologically relevant exposure levels if the critical window for exposure is early in life. Additionally, given that the development of prostate cancer is very slow, length of follow-up in this study might have been insufficient to investigate the association of prostate cancer. However, we previously reported a statistically significant inverse association between plasma isoflavone levels and prostate cancer with the same length of follow-up (Kurahashi et al. 2008b). Third, although selected from the general population, our subjects were restricted to those who participated in the baseline health checkup survey. The incidence in this subcohort was therefore higher than that in total cohort, and any generalization of our results should be considered with caution (Iwasaki et al. 2006). Several strengths also warrant mention. First, blood samples were collected prior to the diagnosis of cancer in our nested case–control study within a population-based prospective study, so the results were not affected by patients’ recall of exposure. Second, cases and controls were selected from the same cohort, thus precluding the possibility of the selection bias inherent to case–control studies.

## Conclusion

This study suggests that no overall association exists between prostate cancer and organochlorines at the levels measured in our study population.

## Figures and Tables

**Table 1:** Baseline characteristics of case and matched control subjects at baseline.

Characteristic	Cases (*n* = 201)	Controls (*n* = 402)	*p*-Value^a^
Mean age ± SD (years)	58.6 ± 6.4	58.4 ± 6.6	—
Body mass index (kg/m^2^) (%)			
< 18.5	2.5	3.5	0.71
18.5–25	72.6	72.1	
≥ 25	24.9	24.4	
Smoking status (%)			
Never	32.8	27.0	0.07
Former	32.8	32.3	
Current	34.4	40.7	
Alcohol intake (≥ 1–2 times/week) (%)	69.2	72.4	0.42
Married (%)	93.5	90.6	0.26
Diet (%)			
Green tea, daily	81.1	80.4	0.97
Miso soup, daily	82.6	78.9	0.18
Milk, daily	44.3	42.8	0.63
Green vegetables, daily	38.3	28.1	0.07
Fish (≥ 3–4 times/week)	55.2	55.0	0.97
Pork (≥ 3–4 times/week)	27.9	27.6	0.28

a *p-*Value for Mantel–Haenszel test with matched-pair strata.

**Table 2:** Plasma levels of lipid-adjusted plasma organochlorines for case and control subjects.

	Cases (*n* = 201)		Controls (*n* = 402)		
	Median (IQR) (ng/g lipid)	< LOD (%)	Median (IQR) (ng/g lipid)	< LOD (%)	*p*-Value^a^
*o,p*′-DDT	4.3 (2.7–7.4)	0	4.3 (2.5–7.7)	0.5	0.99
*p,p*′-DDT	40 (26–63)	0	41 (24–64)	0	0.53
*p,p*′-DDE	910 (560–1600)	0	940 (560–1600)	0	0.38
*trans*-Nonachlor	71 (51–120)	0	75 (50–120)	0	0.89
*cis*-Nonachlor	13 (8.9–21)	0	13 (9.1–21)	0	0.92
Oxychlordane	22 (17–36)	0	24 (17–37)	0	0.32
HCB	54 (32–81)	0	55 (34–85)	0	0.70
Mirex	4.2 (3.0–6.1)	0	4.1 (3.1–6.0)	0	0.47
β-HCH	310 (190–480)	0	320 (200–520)	0	0.57
∑PCBs	425 (312–629)	6.9	448 (319–670)	10.2	0.35
PCB 17	0.8 (0.5–1.2)	26.9	0.9 (0.6–1.3)	29.6	0.53
PCB 28	3.0 (2.8–4.8)	0	3.0 (1.9–4.6)	0	0.27
PCB 51	1.5 (1.0–2.3)	47.8	1.5 (0.9–2.6)	45.3	0.80
PCB 52/69	1.1 (0.8–1.8)	2.5	1.2 (0.8–1.9)	2.5	0.72
PCB 43/49	0.6 (0.5–0.9)	35.3	0.7 (0.5–1.0)	38.1	0.59
PCB 48/47	2.4 (0.9–7.7)	0	3.3 (1.0–8.9)	0	0.81
PCB 44	0.5 (0.4–0.9)	27.4	0.6 (0.4–0.7)	32.1	0.15
PCB 74	14 (8.8–20)	0	14 (9.0–22)	0	0.40
PCB 66	2.7 (1.6–4.5)	0.5	2.5 (1.6–4.3)	0	0.12
PCB 77	22	99.0	ND	100.0	N/A
PCB 90/101	2.6 (1.5–4.2)	0	2.7 (1.7–4.3)	0.2	0.81
PCB 99	14 (9.7–22)	0	15 (10–23)	0	0.22
PCB 123	0.5 (0.4–0.8)	50.2	0.6 (0.5–0.9)	54.2	0.62
PCB 118	24 (15–37)	0	23 (15–38)	0	0.43
PCB 114	1.3 (0.9–1.9)	7.0	1.4 (0.9–2.0)	2.2	0.51
PCB 105	5.0 (3.0–7.7)	0	4.7 (3.0–8.0)	0	0.50
PCB 126	0.4 (0.3–0.7)	90.5	0.4 (0.4–0.5)	94.8	0.19
PCB 146	12 (8.5–18)	0	12 (8.9–19)	0	0.41
PCB 153	96 (70–140)	0	99 (72–150)	0	0.33
PCB 164/163	20 (14–28)	0	20 (15–30)	0	0.56
PCB 138	50 (35–75)	0	51 (38–75)	0	0.20
PCB 128/162	0.7 (0.6–1.2)	15.4	0.8 (0.6–1.1)	16.4	0.77
PCB 167	3.5 (2.6–4.8)	0	3.5 (2.4–5.2)	0	0.76
PCB 156	8.5 (6.4–12)	0	8.7 (6.8–12)	0	0.46
PCB 169	0.5 (0.4–0.6)	98.5	0.5 (0.3–0.6)	99.3	N/A
PCB 182/187	28 (19–44)	0	27 (20–44)	0	0.59
PCB 183	7.3 (5.0–11)	0	7.5 (5.3–11)	0	0.39
PCB 174	0.6 (0.5–0.8)	52.7	0.6 (0.5–0.8)	53.2	0.88
PCB 177	5.2 (3.8–7.5)	0	5.3 (3.8–8.6)	0	0.27
PCB 180	62 (41–95)	0	62 (44–96)	0	0.62
PCB 170	19 (13–28)	0	19 (14–28)	0	0.48
PCB 189	1.0 (0.7–1.5)	2.0	1.0 (0.7–1.4)	2.0	0.73
PCB 202	3.0 (2.0–4.9)	0	2.9 (2.1–4.9)	0	0.69
PCB 201	0.6 (0.5–0.9)	39.8	0.6 (0.5–0.9)	36.3	0.87
PCB 198/199	8.9 (5.9–14)	0	8.8 (6.1–14)	0	0.56
PCB 196	3.0 (1.9–4.2)	0	2.9 (2.0–4.8)	0	0.32
PCB 203	4.6 (3.2–7.5)	0	4.6 (3.2–7.6)	0	0.61
PCB 194	8.8 (5.7–15)	0	8.9 (5.9–15)	0	0.53
PCB 208	1.1 (0.7–1.6)	2.0	1.1 (0.8–1.7)	1.2	0.67
PCB 206	2.5 (1.8–3.8)	0	2.5 (1.8–3.9)	0	0.82
PCB 209	1.8 (1.3–2.4)	0	1.8 (1.3–2.4)	0	0.64

LOD, limit of detection; ND, not detectable. Total PCBs were based on the detectable values of the 41 PCBs included here.

a *p*-Value for Mantel–Haenszel test with matched-pair strata.

**Table 3:** ORs and 95% CIs of prostate cancer, by plasma level of lipid-adjusted organochlorine.

	Quartile category				
Plasma level	Low	Second	Third	High	*p* for trend
*o*,*p*′-DDT (ng/g lipid)	< 2.5	2.5–4.2	4.3–7.6	≥ 7.7	
Cases/controls (*n*)	43/99	57/99	54/101	47/103	
OR (95% CI)	1.00	1.36 (0.81–2.27)	1.27 (0.73–2.20)	1.07 (0.59–1.94)	0.69
OR^a^ (95% CI)	1.00	1.39 (0.79–2.44)	1.29 (0.71–2.34)	1.04 (0.54–2.03)	0.61

*p*,*p*′-DDT (ng/g lipid)	< 24	24–40	41–63	≥ 64	
Cases/controls (*n*)	41/94	64/105	50/101	46/102	
OR (95% CI)	1.00	1.43 (0.86–2.38)	1.14 (0.67–1.96)	1.02 (0.57–1.83)	0.59
OR^a^ (95% CI)	1.00	1.51 (0.87–2.63)	0.92 (0.50–1.70)	1.00 (0.52–1.92)	0.45

*p*,*p*′-DDE (ng/g lipid)	< 560	560–939	940–1599	≥ 1,600	
Cases/controls (*n*)	49/98	52/100	47/94	53/110	
OR (95% CI)	1.00	1.04 (0.64–1.69)	1.00 (0.61–1.64)	0.96 (0.58–1.59)	0.79
OR^a^ (95% CI)	1.00	1.00 (0.60–1.66)	0.89 (0.52–1.53)	0.90 (0.52–1.54)	0.65

*trans*-Nonachlor (ng/g lipid)	< 50	50–74	75–119	≥ 120	
Cases/controls (*n*)	49/96	56/102	44/96	52/108	
OR (95% CI)	1.00	1.07 (0.66–1.75)	0.83 (0.47–1.46)	0.83 (0.43–1.62)	0.54
OR^a^ (95% CI)	1.00	1.11 (0.67–1.86)	0.92 (0.49–1.69)	0.86 (0.42–1.78)	0.61

*cis*-Nonachlor (ng/g lipid)	< 9.1	9.1–12.9	13–20.9	≥ 21	
Cases/controls (*n*)	52/100	48/85	50/111	51/106	
OR (95% CI)	1.00	1.07 (0.66–1.73)	0.83 (0.50–1.38)	0.84 (0.45–1.54)	0.54
OR^a^ (95% CI)	1.00	1.13 (0.67–1.89)	0.83 (0.48–1.43)	0.80 (0.41–1.57)	0.46

Oxychlordane (ng/g lipid)	< 17	17–23.9	24–36.9	≥ 37	
Cases/controls (*n*)	46/89	64/107	42/102	49/104	
OR (95% CI)	1.00	1.14 (0.69–1.88)	0.75 (0.43–1.30)	0.77 (0.39–1.53)	0.32
OR^a^ (95% CI)	1.00	1.06 (0.61–1.82)	0.74 (0.40–1.36)	0.75 (0.34–1.64)	0.40

HCB (ng/g lipid)	< 34	34–54.9	55–84.9	≥ 85	
Cases/controls	54/98	48/102	57/97	42/105	
OR (95% CI)	1.00	0.71 (0.37–1.36)	0.78 (0.38–1.62)	0.49 (0.21–1.12)	0.09
OR^a^ (95% CI)	1.00	0.78 (0.38–1.57)	0.86 (0.40–1.88)	0.52 (0.21–1.25)	0.11

Mirex (ng/g lipid)	< 3.1	3.1–4.0	4.1–5.9	≥ 6.0	
Cases/controls (*n*)	57/97	38/104	50/98	56/103	
OR (95% CI)	1.00	0.59 (0.34–0.996)	0.84 (0.51–1.39)	0.95 (0.54–1.67)	0.66
OR^a^ (95% CI)	1.00	0.54 (0.30–0.97)	0.85 (0.49–1.46)	1.14 (0.61–2.13)	0.22

β-HCH (ng/g lipid)	< 200	200–319	320–519	≥ 520	
Cases/controls (*n*)	52/99	50/97	56/105	43/101	
OR (95% CI)	1.00	0.98 (0.61–1.59)	1.00 (0.62–1.62)	0.78 (0.46–1.33)	0.33
OR^a^ (95% CI)	1.00	0.89 (0.52–1.50)	0.85 (0.50–1.46)	0.56 (0.30–1.01)	0.05

Total PCBs (ng/g lipid)	< 319	319–447	448–668	≥ 669	
Cases/controls (*n*)	67/131	49/89	41/91	44/91	
OR (95% CI)	1.00	1.08 (0.66–1.77)	0.86 (0.51–1.44)	0.89 (0.49–1.61)	0.64
OR^a^ (95% CI)	1.00	1.06 (0.63–1.79)	0.84 (0.49–1.46)	0.97 (0.51–1.87)	0.90

a Adjusted for smoking status, alcohol intake, marital status, body mass index, and intake of green tea and miso soup.

Adjusted ORs were calculated based on subjects with complete information on covariates.

**Table 4:** ORs and 95% CIs of prostate cancer according to plasma level of lipid-adjusted organochlorines by stage.

	Quartile category				
Organochlorine	Low	Second	Third	High	*p* for trend
Localized					
*o*,*p*′-DDT					
Cases/controls	25/66	41/70	44/70	29/72	
OR^a^ (95% CI)	1.00	1.79 (0.88–3.65)	2.01 (0.96–4.22)	1.17 (0.50–2.74)	0.59

*p*,*p*′-DDT					
Cases/controls	30/65	46/71	37/71	26/71	
OR^a^ (95% CI)	1.00	1.35 (0.71–2.55)	0.97 (0.49–1.93)	0.65 (0.29–1.46)	0.12

*p*,*p*-DDE					
Cases/controls	35/67	34/70	48/70	22/71	
OR^a^ (95% CI)	1.00	0.86 (0.47–1.59)	1.33 (0.72–2.45)	0.56 (0.27–1.15)	0.14

*trans*-Nonachlor					
Cases/controls	35/67	42/67	28/75	34/69	
OR^a^ (95% CI)	1.00	1.18 (0.65–2.14)	0.57 (0.26–1.25)	0.74 (0.29–1.90)	0.65

*cis*-Nonachlor					
Cases/controls	35/69	37/61	34/77	33/71	
OR^a^ (95% CI)	1.00	1.22 (0.67–2.24)	0.82 (0.42–1.61)	0.86 (0.38–1.95)	0.65

Oxychlordane					
Cases/controls	31/63	48/74	26/71	34/70	
OR^a^ (95% CI)	1.00	1.19 (0.62–2.30)	0.70 (0.33–1.46)	0.80 (0.30–2.10)	0.53

HCB					
Cases/controls	36/66	29/70	43/71	31/71	
OR^a^ (95% CI)	1.00	0.72 (0.28–1.87)	0.97 (0.37–2.53)	0.73 (0.26–2.06)	0.64

Mirex					
Cases/controls	33/67	29/66	44/77	33/68	
OR^a^ (95% CI)	1.00	0.90 (0.45–1.81)	1.21 (0.63–2.32)	1.27 (0.58–2.78)	0.44

β-HCH					
Cases/controls	35/63	32/69	42/75	30/71	
OR^a^ (95% CI)	1.00	0.85 (0.44–1.63)	1.10 (0.59–2.06)	0.69 (0.34–1.37)	0.33

∑PCBs					
Cases/controls	41/88	33/64	35/63	30/63	
OR^a^ (95% CI)	1.00	1.24 (0.66–2.34)	1.33 (0.71–2.50)	1.32 (0.59–2.96)	0.59

Advanced					
*o*,*p*′-DDT					
Cases/controls	10/24	12/25	11/23	11/23	
OR^a^ (95% CI)	1.00	0.81 (0.25–2.63)	0.75 (0.18–3.18)	0.96 (0.22–4.26)	0.93

*p*,*p*′-DDT					
Cases/controls	8/21	12/26	15/23	9/25	
OR^a^ (95% CI)	1.00	0.99 (0.27–3.69)	1.42 (0.34–5.99)	0.61 (0.11–3.38)	0.55

*p*,*p*′-DDE					
Cases/controls	13/23	11/24	5/21	15/27	
OR^a^ (95% CI)	1.00	0.98 (0.29–3.36)	0.23 (0.05–1.18)	0.69 (0.19–2.55)	0.64

*trans*-Nonachlor					
Cases/controls	10/23	11/24	9/22	14/26	
OR^a^ (95% CI)	1.00	1.12 (0.31–4.10)	1.19 (0.29–4.88)	2.46 (0.44–13.73)	0.28

*cis*-Nonachlor					
Cases/controls	13/24	7/20	10/27	14/24	
OR^a^ (95% CI)	1.00	0.48 (0.12–1.85)	0.71 (0.21–2.39)	1.55 (0.34–7.11)	0.43

Oxychlordane					
Cases/controls	12/23	11/24	13/24	8/24	
OR^a^ (95% CI)	1.00	0.50 (0.13–2.01)	0.78 (0.19–3.27)	0.31 (0.04–2.54)	0.38

HCB					
Cases/controls	10/24	11/22	10/25	13/24	
OR^a^ (95% CI)	1.00	1.13 (0.24–5.21)	0.53 (0.11–2.43)	0.78 (0.10–6.10)	0.90

Mirex					
Cases/controls	14/21	8/27	7/24	15/23	
OR^a^ (95% CI)	1.00	0.38 (0.10–1.46)	0.38 (0.10–1.49)	1.60 (0.35–7.23)	0.26

β-HCH					
Cases/controls	12/23	14/24	7/24	11/24	
OR^a^ (95% CI)	1.00	0.64 (0.19–2.12)	0.35 (0.07–1.69)	0.31 (0.07–1.45)	0.23

∑PCBs
Cases/controls	18/27	9/23	6/22	11/23	
OR^a^ (95% CI)	1.00	0.36 (0.09–1.49)	0.18 (0.04–0.92)	0.32 (0.06–1.61)	0.17

a Adjusted for smoking status, alcohol intake, marital status, body mass index, and intake of green tea and miso soup.

Adjusted ORs were calculated based on subjects with complete information on covariates.

## Appendix I

Members of the JPHC Study Group (principal investigator: S. Tsugane): M. Inoue, T. Sobue, and T. Hanaoka, Research Center for Cancer Prevention and Screening, National Cancer Center, Tokyo; J. Ogata, S. Baba, T. Mannami, A. Okayama, and Y. Kokubo, National Cardiovascular Center, Suita; K. Miyakawa, F. Saito, A. Koizumi, Y. Sano, I. Hashimoto, T. Ikuta, and Y. Tanaba, Iwate Prefectural Ninohe Public Health Center, Ninohe; Y. Miyajima, N. Suzuki, S. Nagasawa, Y. Furusugi, and N. Nagai, Akita Prefectural Yokote Public Health Center, Yokote; H. Sanada, Y. Hatayama, F. Kobayashi, H. Uchino, Y. Shirai, T. Kondo, R. Sasaki, Y. Watanabe, Y. Miyagawa, Y. Kobayashi, and M. Machida, Nagano Prefectural Saku Public Health Center, Saku; Y. Kishimoto, E. Takara, T. Fukuyama, M. Kinjo, M. Irei, and H. Sakiyama, Okinawa Prefectural Chubu Public Health Center, Okinawa; K. Imoto, H. Yazawa, T. Seo, A. Seiko, F. Ito, F. Shoji, and R. Saito, Katsushika Public Health Center, Tokyo; A. Murata, K. Minato, K. Motegi, and T. Fujieda, Ibaraki Prefectural Mito Public Health Center, Mito; T. Abe, M. Katagiri, M. Suzuki, and K. Matsui, Niigata Prefectural Kashiwazaki and Nagaoka Public Health Center, Kashiwazaki and Nagaoka; M. Doi, A. Terao, Y. Ishikawa, and T. Tagami, Kochi Prefectural Chuo-higashi Public Health Center, Tosayamada; H. Doi, M. Urata, N. Okamoto, F. Ide, and H. Sueta, Nagasaki Prefectural Kamigoto Public Health Center, Arikawa; H. Sakiyama, N. Onga, H. Takaesu, and M. Uehara, Okinawa Prefectural Miyako Public Health Center, Hirara; F. Horii, I. Asano, H. Yamaguchi, K. Aoki, S. Maruyama, M. Ichii, and M. Takano, Osaka Prefectural Suita Public Health Center, Suita; S. Matsushima and S. Natsukawa, Saku General Hospital, Usuda; M. Akabane, Tokyo University of Agriculture, Tokyo; M. Konishi, K. Okada, and I. Saito, Ehime University, Toon; H. Iso, Osaka University, Suita; Y. Honda, K. Yamagishi, S. Sakurai, and N. Tsuchiya, Tsukuba University, Tsukuba; H. Sugimura, Hamamatsu University, Hamamatsu; Y. Tsubono, Tohoku University, Sendai; M. Kabuto, National Institute for Environmental Studies, Tsukuba; S. Tominaga, Aichi Cancer Center Research Institute, Nagoya; M. Iida, W. Ajiki, and A. Ioka, Osaka Medical Center for Cancer and Cardiovascular Disease, Osaka; S. Sato, Osaka Medical Center for Health Science and Promotion, Osaka; N. Yasuda, Kochi University, Nankoku; K. Nakamura, Niigata University, Niigata; S. Kono, Kyushu University, Fukuoka; K. Suzuki, Research Institute for Brain and Blood Vessels Akita, Akita; Y. Takashima and M. Yoshida, Kyorin University, Mitaka; E. Maruyama, Kobe University, Kobe; M. Yamaguchi, Y. Matsumura, S. Sasaki, and S. Watanabe, National Institute of Health and Nutrition, Tokyo; T. Kadowaki, Tokyo University, Tokyo; M. Noda and T. Mizoue, International Medical Center of Japan, Tokyo; Y. Kawaguchi, Tokyo Medical and Dental University, Tokyo; and H. Shimizu, Sakihae Institute, Gifu.
